# Molecular Characterization and Antibiotic Resistance of Avian Pathogenic *Escherichia coli* (APEC) Isolates from Broiler Chickens in Algeria

**DOI:** 10.3390/ani15223324

**Published:** 2025-11-18

**Authors:** Ismail Boulbair, Jiangang Hu, Abdelhamid Hammoudi, Beibei Zhang, Saad Aissat, Xinyu Wang, Mohammed Foudil, Shaohui Wang

**Affiliations:** 1 Institute of Veterinary Sciences, University of Tiaret, Tiaret 14000, Algeria; hammoudiabdelhamid@yahoo.fr (A.H.); aissat.saad@gmail.com (S.A.); foudilveto@gmail.com (M.F.); 2 Laboratory of Hygiene and Animal Pathology, University of Tiaret, Tiaret 14000, Algeria; 3 Shanghai Veterinary Research Institute, Chinese Academy of Agricultural Sciences, Shanghai 200241, China; hjg@shvri.ac.cn (J.H.); bzhang1997@sina.com (B.Z.); wangxinyu1823@163.com (X.W.); 4 Laboratory of Research on Local Animal Products, University of Tiaret, Tiaret 14000, Algeria; 5 Laboratory of Nutrition, Biodiversity and Environment, University of Medea, Medea 26000, Algeria

**Keywords:** colibacillosis, APEC, serotypes, virulence genes, phylogenetic groups, antibiotic resistance

## Abstract

Avian pathogenic *Escherichia coli* (APEC) causes avian colibacillosis, a disease responsible for high morbidity and mortality in commercial poultry flocks, leading to devastating economic losses to the poultry industry worldwide. APEC may also act as a source of virulence and antibiotic resistance genes that can be transferred to other *Escherichia coli* pathotypes. Therefore, this study aimed to determine the serotypes, phylogenetic background, virulence genes, and antibiotic resistance profiles of APEC in Algeria. A total of 98 APEC strains were isolated from chicken samples with characteristic colibacillosis signs between 2019 and 2020. O-serotyping identified O157 (20.41%) and O78 (11.22%) as the predominant serotypes. The isolates were classified into groups B1 (43.87%), C (29.59%), A (12.24%), E (7.14%), F (5.10%), and B2 (2.04%). Virulence gene analysis revealed that among the 31 genes investigated, a high occurrence of *mat*, *crlA* (100% each), *yijP* (98.98%), *fimC*, *ibeB*, *ompA* (97.96% each), *iucD* (89.80%), *iroN* (81.63%), *iss* (80.61%), and *eae* (79.59%) was observed. The highest resistance rates were found for ampicillin (97.96%), amoxicillin–clavulanic acid (96.94%), nalidixic acid (94.90%), tetracycline (90.82%), and ciprofloxacin (79.59%). Additionally, 92.86% of APEC isolates were resistant to three or more antibiotics, reflecting extensive antimicrobial use in Algerian poultry farms and highlighting a major challenge for animal health management and a potential risk of zoonotic transmission. Our data provide valuable insights into the characteristics of the APEC populations in broiler chickens in Algeria. This may assist in understanding APEC pathogenesis and in developing effective control strategies.

## Simple Summary

Avian pathogenic *Escherichia coli* (APEC) is a significant pathogen that affects commercial poultry and leads to substantial financial losses worldwide. These bacteria utilize various strategies to infect avian hosts and can pose a potential public health threat. In this study, APEC isolates collected from broiler chickens in Algeria were analyzed for their serotypes, virulence genes, phylogenetic groups, and resistance to antibiotics. Notably, this study is the first to report that the O157 serotype, typically associated with human diseases, may represent an emerging serotype within the APEC population in Algeria. The study also revealed a variable distribution of virulence genes and phylogenetic groups among the isolates. Furthermore, most isolates showed resistance to antimicrobials commonly used in avian medicine, with a high proportion exhibiting multi-antibiotic resistance. These findings emphasize the necessity for regular monitoring of these harmful, antibiotic-resistant bacteria, which allows the early detection and implementation of control measures to limit their spread in poultry and prevent transfer to humans.

## 1. Introduction

Avian pathogenic *Escherichia coli* (APEC), a subgroup within the pathovar of extraintestinal pathogenic *Escherichia coli* (ExPEC), is the causative organism of both localized and systemic infections affecting a wide range of avian species, including chickens, turkeys, and ducks [1]. APEC infections can manifest in various forms, such as airsacculitis (chronic respiratory disease), colisepticemia, and omphalitis/yolk sac infection, collectively termed avian colibacillosis [2]. This disease causes enormous economic losses to the avian industry worldwide, primarily due to high mortality rates and reduced productivity in terms of growth, hatching, and egg-laying, as well as carcass seizures at slaughterhouses and expenses associated with treatment and prophylactic measures [3].

APEC strains possess a range of genes encoding virulence factors that enable them to establish infection in avian hosts, including adhesins, invasins, iron uptake systems, protectins, and toxins [1]. These factors contribute to bacterial adhesion, invasion, colonization, replication, and host cell damage, as well as evasion of the host’s immune defenses [4]. Several studies have identified various combinations of virulence genes to define the APEC pathotype and distinguish it from commensal avian fecal *Escherichia coli* (AFEC). However, this remains complex, and characterizing the APEC pathotype continues to be challenging [5,6,7,8]. It is widely accepted that APEC represents a group of *Escherichia coli* (*E. coli*) with multiple distinct genotypes, capable of acting as both primary and opportunistic pathogens [3,9].

Phylogenetic typing remains a valuable method not only to characterize *E. coli* populations but also to highlight the link between phylotypes and virulence potential, as well as diseases caused by this organism. Clermont and colleagues described a phylogenetic approach to categorize *E. coli* into eight phylogenetic groups, including *E. coli* sensu stricto A, B1, B2, C, D, E, F, and *E. coli* cryptic clade I [10]. Logue et al. [11] reported that the majority of human ExPEC strains belonged to phylogroups B2 and D, whereas most APEC were classified in groups C, F, and B1.

Although APEC is one of the major bacterial pathogens affecting poultry, only a limited number of serotypes, such as O1, O2, and O78, have been commonly associated with the disease outbreaks [1].

For several decades, antibiotics have been widely used as prophylactic and therapeutic agents to combat infections caused by APEC [12]. The widespread irrational use of antibiotics in poultry farming has led to the selection and dissemination of resistant and/or multidrug-resistant (MDR) *E. coli* [13]. In addition to treatment failures and the resulting economic losses in poultry production, these resistant pathogens can be transmitted to humans through the food chain, contaminated environments, or direct contact with infected birds, posing significant challenges to public health [14,15,16]. It has been demonstrated that certain APEC strains share genetic similarities and a comparable virulence gene content with human ExPEC, particularly uropathogenic *E. coli* (UPEC) and neonatal meningitis *E. coli* (NMEC) [1]. This overlap and evidence of experimental cross-infections involving avian hosts and animal models of human infections suggest that APEC may act as zoonotic agents [9].

Poultry production in Algeria has expanded rapidly over the past decades to meet growing protein demand, particularly in urban areas, with intensive farming systems becoming increasingly common [17]. However, poor management practices in these intensive systems have increased the risk of diseases such as colibacillosis [18], resulting in antibiotic misuse and, thereby, facilitating the emergence of antimicrobial resistance among poultry-associated bacteria, including APEC, as recently reported by Kamel et al. [19] and Chenouf et al. [20]. Despite the importance of APEC and the risk of antimicrobial resistance in Algerian poultry, data on the characteristics as well as the antibiotic resistance of APEC remain limited [20,21]. Therefore, the objective of the present study was to investigate the serotypes, virulence genes, phylogenetic groups, and antimicrobial susceptibility patterns of APEC isolates from diseased broiler chickens.

## 2. Materials and Methods

### 2.1. Sampling, E. coli Isolation, and Identification

A total of 120 organ (heart, liver, spleen, and yolk sac) samples were collected between January 2019 and August 2020 from diseased and dead broiler chickens with characteristic clinical signs and pathological lesions of colibacillosis. These broilers originated from different commercial poultry farms located in Algeria.

Swabs from organs were aseptically streaked on MacConkey agar and incubated aerobically at 37 °C for 18–24 h. Suspect bacterial colonies were sub-cultured onto methylene blue (EMB) agar and incubated overnight aerobically at 37 °C, and the green metallic sheen suspected colony was picked for further characterization.

The isolates with typical morphological characteristics were subsequently subjected to the Gram stain, the oxidase test, and biochemical identification with API^®^ 20E commercial kits (bioMérieux, Marcy-l’Étoile, France) according to the manufacturer’s instructions. *E. coli* isolates were also confirmed by polymerase chain reaction (PCR) identification for the *E. coli* alkaline phosphatase *phoA* gene [22]. All *E. coli* isolates were stored in Luria–Bertani (LB) broth containing 50% glycerol at −80 °C until further analysis.

### 2.2. DNA Extraction

For the PCR assays, bacterial DNA from each isolate was extracted using the boiling method as previously reported [20]. In brief, bacterial cultures were collected by centrifugation and resuspended in nuclease-free water. The bacterial suspensions were then boiled at 100 °C for 10 min. After centrifuging the bacterial lysates, the supernatants were collected and used as DNA templates.

### 2.3. Serotyping

Conventional agglutination technique and O-genotyping PCR were used to identify the O-serotypes as previously described [23]. The serotyping was performed by targeting genes involved in O-antigen biosynthesis using primers as described previously, with details provided in Table S1 [24,25]. The serotypes of APEC isolates were confirmed through a serum agglutination test using monovalent O antisera (Statens Serum Institute, Copenhagen, Denmark) according to the protocol provided by the manufacturer.

### 2.4. Phylogenetic Analysis

The phylogenetic classification of the APEC isolates was determined using a quadruplex PCR protocol for simultaneously detecting the following genes: *chuA*, *yjaA*, *arpA*, and the DNA fragment *TspE4.C2*, as previously reported [10]. *E. coli* isolates were categorized into eight distinct phylogroups (A, B1, B2, C, D, E, F, and clade I) according to the PCR assay results.

### 2.5. PCR-Based Screening of Virulence Genes

APEC isolates were investigated for the presence of virulence genes by multiplex PCR as described previously [26]. The multiplex PCR assays were designed to simultaneously detect the following virulence genes: 1 (*fimC*, *papC*, *tsh*, *mat*, *aatA*); 2 (*ibeA*, *ibeB*, *yijp*, *vat*); 3 (*iss*, *cva/cvi*, *ompA*, *neuC*); 4 (*chuA*, *fyuA*, *irp2*, *iucD*, *iroN*); 5 (*iha*, *cnf1/2*); 6 (*stx1*, *F17b*); 7 (*afa/draB*, *crlA*); 8 (*hlyA*, *STb*); 9 (*LT*, *STa*); 10 (*stx2*, *eae*, *F17c*).

All primers used in the amplification procedures were synthesized by Sangon Biotech (Shanghai, China), and their details are listed in Table S1 [26,27,28,29,30,31,32]. PCR amplifications were performed in a 20 μL reaction system as per the manufacturer’s instructions (Vazyme Biotech Co., Ltd., Nanjing, China). APEC strains O18, O78, DE719, and *E. coli* O157 were employed as positive controls in PCR experiments [24,30,33], whereas sterile distilled water was included as the negative control. The amplified PCR products were analyzed by electrophoresis on a 1% agarose gel, visualized under UV light, and compared with the DL2000 molecular size marker (Vazyme Biotech Co., Ltd., Nanjing, China).

### 2.6. Antimicrobial Susceptibility Assessment

The Kirby–Bauer disk diffusion method on Mueller–Hinton agar was performed to test the antibiotic sensitivity of the isolates using the following commercially available antibiotics: ampicillin (10 μg), amoxicillin–clavulanic acid (20/10 μg), cefotaxime (30 μg), gentamicin (10 μg), kanamycin (30 μg), tetracycline (30 μg), ciprofloxacin (5 μg), nalidixic acid (30 μg), trimethoprim–sulphamethoxazole (1.25/23.75 μg), and chloramphenicol (30 μg). These antibiotics belonged to six different classes and were chosen based on their common use in clinical practice or their importance in epidemiological surveillance. All inhibition zone diameter results were recorded, and isolates were classified as susceptible (S), intermediate (I), and resistant (R) according to the Clinical and Laboratory Standards Institute (CLSI) guidelines [34]. *E. coli* ATCC 25922 served as the quality control strain.

### 2.7. Statistical Analysis

Data analysis was performed using Microsoft Excel 2021, and graphs were generated using GraphPad Prism (version 10.5.0) for Windows (GraphPad Software, San Diego, CA, USA).

## 3. Results

### 3.1. Isolation and Confirmation of E. coli

Out of 120 organ samples collected for bacteriological examination, 98 isolates (81.66%) were identified as *E. coli* using the API^®^ 20E system and PCR.

### 3.2. Serotype Determination

O-genotyping PCR and serum agglutination techniques showed that a large proportion of the APEC isolates (65.31%, 64/98) were of unidentified serotypes, whereas (34.69%, 34/98) were typeable.

**Figure 1:**
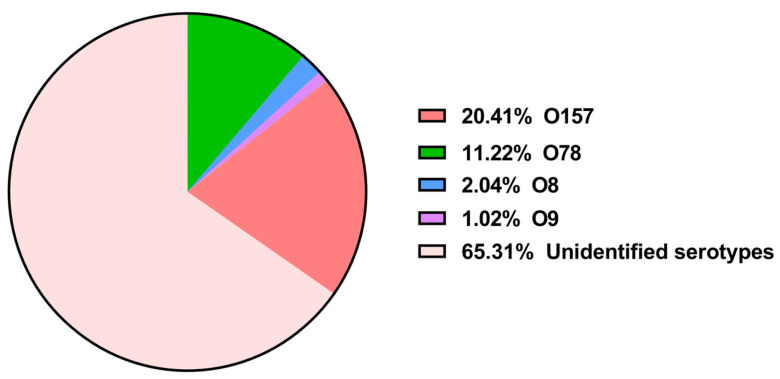
Serotype classification of the 98 APEC isolates.

Among the typeable APEC isolates, four O-serotypes were identified, with O157 being the most frequently observed (20.41%, 20/98), followed by O78 (11.22%, 11/98). Other serotypes, such as O8 and O9, were uncommon, accounting for (2.04%, 2/98) and (1.02%, 1/98), respectively. None of the isolates belonged to the O1, O2, O18, or O149 serotype (Figure 1 and Table S2).

### 3.3. Identification of APEC Phylogenetic Groups

**Figure 2:**
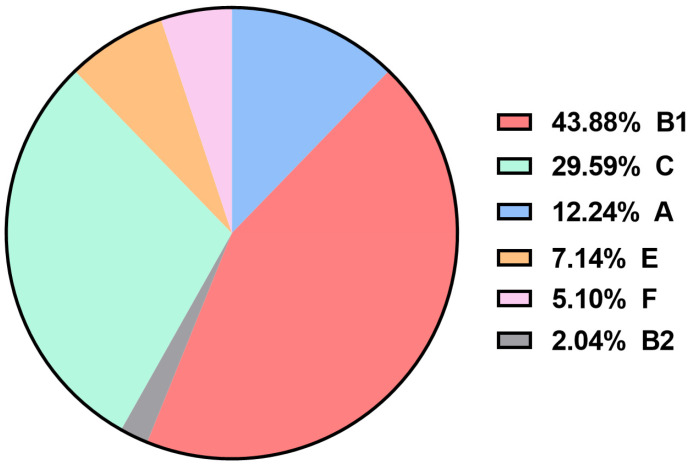
Phylogenetic group composition of APEC isolates.

Phylogenetic analysis based on the novel Clermont’s phylogenetic typing scheme showed that most of the APEC isolates belonged to phylogroup B1 (43.88%, 43/98), C (29.59%, 29/98), and A (12.24%, 12/98). Only (7.14%, 7/98) of the APEC isolates belonged to phylogroup E, (5.10%, 5/98) to phylogroup F, and (2.04%, 2/98) to phylogroup B2, while none of the APEC isolates belonged to phylogroup D or clade I (Figure 2 and Table S3).

### 3.4. Virulence Gene Detection and Distribution

**Figure 3:**
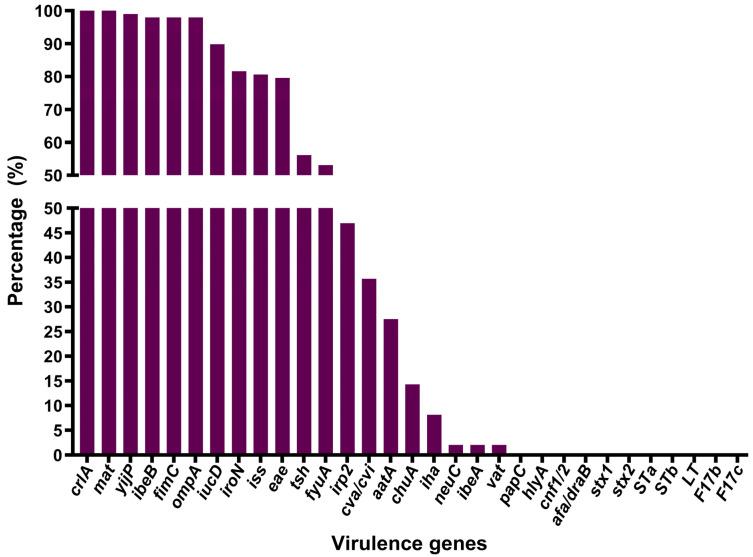
Percentage distribution of virulence genes in APEC isolates.

The virulence genotyping showed that *mat* and *crlA* genes were present in all 98 (100%) APEC isolates. The *yijP* gene was detected in 97 (98.98%) isolates. *fimC*, *ibeB*, and *ompA* were found in 96 (97.96%) isolates. The *iucD*, *iroN*, *iss*, and *eae* genes were present in 88 (89.80%), 80 (81.63%), 79 (80.61%), and 78 (79.59%) isolates, respectively. *tsh*, *fyuA*, *irp2*, *cva/cvi*, and *aatA* were identified in 55 (56.12%), 52 (53.06%), 46 (46.94%), 35 (35.71%), and 27 (27.55%) isolates, respectively. Additionally, 14 (14.29%) isolates tested positive for *chuA* and 8 (8.16%) for *iha*, while *ibeA*, *vat*, and *neuC* were only detected in 2 (2.04%) isolates. None of the APEC isolates carried *papC*, *cnf1/2*, *stx1*, *stx2*, *F17b*, *F17c*, *hlyA*, *STa*, *STb*, *LT*, or *afa/draB* genes (Figure 3 and Table S4). All APEC isolates in this study contained at least eight virulence genes.

### 3.5. Distribution of Virulence Genes Across Serotypes and Phylogenetic Groups

**Figure 4:**
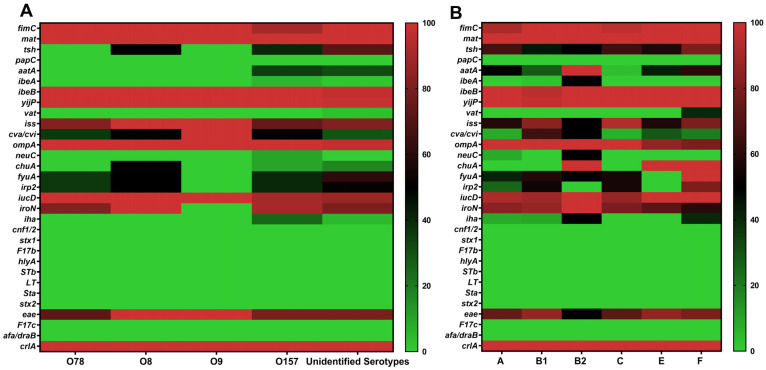
Distribution of virulence genes according to serotypes and phylogenetic groups. (**A**) Virulence gene percentages across identified serotypes. (**B**) Distribution of virulence genes based on phylogenetic groups.

The relationship between virulence genes and serotypes was examined (Table 1 and Figure 4A). The *fimC*, *ibeB*, *yijP*, *iss*, *ompA*, *iucD*, *eae*, and *crlA* genes showed a similar distribution among serotypes O157, O78, O8, O9, and unidentified serotype isolates. The *cva/cvi* gene had relatively similar percentages in O157, O78, O8, and the unidentified serotypes was also found in the single O9 isolate. The *mat*, *iroN*, *fyuA*, and *irp2* genes, although present at comparable frequencies in serotypes O157, O78, O8, and unidentified serotype isolates, were absent in O9. Among the studied genes, *aatA* had a comparable detection rate in O157 (35%) and unidentified serotype isolates (31.25%), but it was completely absent in O78, O8, and O9. The *chuA* gene was detected in 50% of O8 isolates; in contrast, it was found in 10% of O157 and 17.19% of unidentified serotype isolates and not in O78 and O9. The *iha* gene was present in 25% of O157 isolates; however, its prevalence in unidentified serotype isolates was much lower (4.69%), and it was not detected in O78, O8, or O9. The *neuC* gene was only found in the O157 serotype (10%) and not detected in O78, O8, O9, or unidentified serotype isolates. The *ibeA* gene was present in a single O157 isolate (5%) and in one unidentified serotype isolate (1.56%), but not in O78, O8, or O9. The *vat* gene was absent in all defined serotypes (O157, O78, O8, and O9), with detection only in 3.13% of unidentified serotype isolates. The *tsh* gene was found in 40% of O157 isolates and was more common in unidentified serotype isolates (over 70%). It was detected in 50% of O8 isolates but was absent in O78 and O9 (Table 1 and Figure 4A).

The association between virulence genes and the identified phylogenetic groups was investigated (Table 2 and Figure 4B). The *crlA*, *fimC*, *mat*, *ibeB*, *yijP*, and *iucD* genes were significantly distributed across all phylogroups. In contrast, the *iha*, *iroN*, *ibeA*, *neuC*, and *aatA* genes were predominantly detected in phylogroup B2, with frequencies of either 50% or 100%, compared to the other groups. The *vat* gene was exclusively identified in phylogroup F (40%). Among the iron acquisition-related genes, *chuA* was widely distributed in phylogroups B2, E, and F (100% each), while *irp2* and *fyuA* were highly prevalent in phylogroup F (80% and 100%, respectively). The intimin-encoding gene *eae* was less frequently detected in phylogroup B2 than in the other groups. The *ompA* gene was universally present in phylogroups A, B1, B2, and C (100% each), and slightly less prevalent in groups E and F (85.71% and 80%, respectively). The *iss* gene was strongly associated with isolates from phylogroups C, B1, and F, with respective frequencies of 93.10%, 83.72%, and 80%, compared to lower frequencies observed in the remaining groups. *tsh* had the highest level in group F (80%) and showed comparable percentages in groups A (66.67%), C (65.52%), E (57.14%), B1 (44.19%), and B2 (50%), while *cva/cvi* were mainly associated with phylogroup B1 (Table 2 and Figure 4B).

Notably, the co-occurrence of some pivotal virulence genes was observed in phylogroups. In phylogroup B2, one isolate, out of a total of two identified in this group, simultaneously carried *neuC* and *ibeA*. Similarly, in phylogroup F, two isolates harbored a combination of *fyuA*, *chuA*, *irp2*, and *vat* genes (Table 2 and Table S7).

As shown in Table 2, phylogenetic group F had the highest mean virulence score (MVS) of 14.20, followed closely by phylogenetic group B2 with an MVS of 13.50, while phylogenetic groups A, B1, C, and E showed comparable MVS, ranging from 11.08 to 11.97. The MVS for each phylogenetic group was calculated by dividing the total number of virulence genes identified in isolates of the same group (excluding the diarrheagenic gene *eae*) by the number of isolates within that group, as described by El-Shaer et al. [35].

### 3.6. Antibiotic Susceptibility Testing of APEC

**Figure 5:**
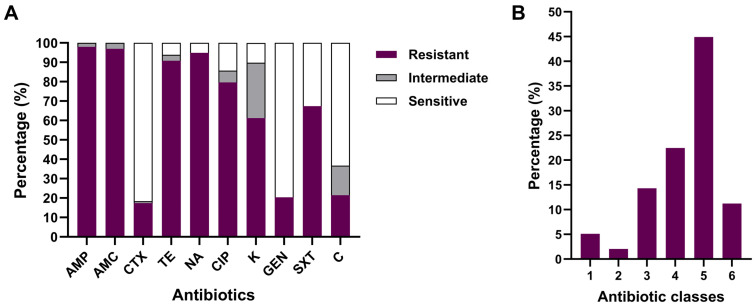
Results of antimicrobial sensitivity testing (**A**) Frequencies of APEC isolates according to their antibiotic susceptibility patterns. AMP: Ampicillin, AMC: Amoxicillin–clavulanic acid, CTX: Cefotaxime, TE: Tetracycline, NA: Nalidixic acid, CIP: Ciprofloxacin, K: Kanamycin, GEN: Gentamycin, SXT: Trimethoprim–Sulfamethoxazole, C: Chloramphenicol. (**B**) Proportion of Multidrug-Resistant (MDR) APEC isolates.

Our results indicated high levels of resistance toward ampicillin (97.96%), amoxicillin–clavulanic acid (96.94%), nalidixic acid (94.90%), tetracycline (90.82%), and ciprofloxacin (79.59%). Moderate resistance rates of 67.35% and 61.22% were recorded for trimethoprim–sulfamethoxazole and kanamycin, respectively, whereas low frequencies of resistance to chloramphenicol (21.40%), gentamicin (20.40%), and cefotaxime (17.30%) were observed (Figure 5A and Table S5). In addition, 91 isolates (92.86%) were resistant to at least three different families of antibiotics and consequently classified as multidrug-resistant (MDR) according to the criteria of Sweeney et al. [36]. The proportion of the APEC isolates that exhibited resistance to 5, 4, and 3 antibiotic categories was 44 (44.90%), 22 (22.45%), and 14 (14.29%), respectively, while 11 (11.22%), 5 (5.10%), and 2 (2.04%) of the APEC isolates were resistant to a panel of 6, 1, and 2 antibiotic classes (Figure 5B and Table S6), respectively.

## 4. Discussion

Our findings showed that the majority of the isolates were of unidentified serotypes (64%). Previous studies conducted in Algeria and elsewhere have also reported a high frequency of unidentified serotypes of APEC isolates [37,38].

Unexpectedly, among the typeable isolates, O157 was the most frequently observed serotype, accounting for 20.41% of the tested APEC isolates. In Albania, *E. coli* O157 has been reported at a low prevalence among APEC isolates from birds affected by colibacillosis (7.67%) [39], whereas in Egypt, O157 has recently been identified as one of the predominant serotypes, with a prevalence similar to that observed in our study (20%) [40]. The pathogenicity of this serotype has been experimentally demonstrated in one-day-old chicks [41]. The O157 serotype is classically associated with Shiga-toxin-producing *E. coli* (STEC O157:H7), one of the most notorious foodborne pathogens causing severe human diseases such as hemorrhagic colitis and hemolytic uremic syndrome (HUS) [42]. The detection of serotype O157 in avian *E*. *coli* isolates suggests that poultry may serve as a potential zoonotic reservoir capable of transmitting these strains to humans [43]. 

None of the 98 APEC isolates were identified as belonging to serotypes O1 or O2, which is in accordance with previous reports from Egypt [44]. In contrast, a recent study conducted in northeast Algeria [20] demonstrated that serotypes O1 and O2 were detected in 31.30% and 33.20% of the isolates, respectively. The observed regional differences in Algeria suggest that APEC serotype distribution may be influenced by variations in poultry farming practices and local biosecurity measures. Furthermore, serotype O78 was observed at a prevalence of 11.22% in our study. This finding aligns with results obtained in Algeria and Poland, where approximately 14% of the isolates belonged to this serotype [20,45]. O78 has been widely reported as the most common serotype among APEC isolates in several countries [13,46,47]. Other serotypes identified in our study, such as O8 and O9, have also been documented in earlier research [45,48].

The analysis of the distribution of virulence genes among serotypes revealed that O157 possessed a multitude of such genes, several of which were also present in the classical APEC serotype O78. This suggests that the O157 serotype, most commonly associated with cattle, may represent an emerging serotype of APEC in Algeria, reflecting possible interspecies transmission dynamics within local poultry ecosystems. Similarly, O145, a serotype typically linked to Shiga-toxin-producing *E. coli* (STEC), has been identified as an APEC serotype of concern in China [49].

Our results, together with data from Egypt, suggest a changing trend within the population of disease-causing APEC serotypes in North African poultry production systems, likely driven by regional factors, and warrant special attention and continuous monitoring.

The species *E. coli* comprises eight distinct phylogenetic groups (A, B1, B2, C, D, E, F, and clade I) [10]. In the present study, phylogenetic analysis revealed a significant predominance of groups B1 and C, followed by A, E, F, and B2. These findings align with recent studies from South Korea and Brazil, which reported that most APEC isolates predominantly belong to phylogenetic group B1 [50,51]. A notable proportion (65%) of APEC isolates associated with this group were positive for the ColV plasmid, as confirmed by the presence of *cva/cvi* genes. These results indicate that, even in strains belonging to phylogenetic groups other than B2, the acquisition of virulent plasmids such as ColV can increase their ability to colonize extraintestinal sites and cause avian colibacillosis [5].

Previous investigations have shown that the distribution of phylogenetic groups among APEC populations varies in predominance between countries [11,51,52]. It appears that APEC differs from human ExPEC in terms of phylogenetic classification [11]. This difference may be explained by the fact that virulence genes essential for APEC pathogenesis are mainly plasmid-borne; consequently, Clermont’s phylogenetic grouping based on a limited number of chromosomal markers is often an inadequate method for accurately identifying the APEC pathotype [53].

APEC possesses diverse iron acquisition systems, including siderophores (yersiniabactin, aerobactin, salmochelin) as well as transporters to capture iron from the host’s body fluids [54]. These siderophores and transporters not only contribute to iron acquisition but also participate in APEC adhesion, invasion, the expression of additional virulence genes, protection from environmental stresses, colonization, and persistence in the host [1]. A significant proportion of the APEC isolates analyzed in this study harbored genes involved in iron scavenging mechanisms, such as *iucD*, *iroN*, *fyuA*, and *irp2*, with frequencies of 89.80%, 81.63%, 53.06%, and 46.94%, respectively. These findings are relatively comparable to reports from earlier studies conducted in China and Portugal [48,55]. In prior studies, the *iucD*, *iroN*, *fyuA*, and *irp2* genes were reported to be more prevalent in APEC isolates than in AFEC isolates [8,55].

APEC isolates also exhibited a notable prevalence of adhesin genes, specifically *mat* (100%), *crlA* (100%), *fimC* (97.96%), and *tsh* (56.12%), whereas *aatA* was detected less frequently, with a prevalence of 27.55%. These findings align with previous research on ExPEC associated with Mink Hemorrhagic Pneumonia in China [56], which reported frequencies of 89.40%, 96.50%, and 15.3% for the *mat*, *fimC*, and *aatA* genes, respectively. In line with our data, Al-Kandari and Woodward [57] reported that the *crlA* gene, involved in regulating the curli fimbrial operon, was present in all APEC isolates (100%). Furthermore, Awawdeh et al. [58] observed a comparable prevalence of the *tsh* gene (55%) in Australia.

It is noteworthy that, in contrast to a previous study conducted in Algeria [15], which reported that all APEC isolates tested negative for the *eae* gene, our findings revealed a significantly higher prevalence (79.59%). The *eae* gene, located within a pathogenicity island known as the Locus of Enterocyte Effacement (LEE) in the genomes of Enteropathogenic *E. coli* (EPEC) and Enterohemorrhagic *E. coli* (EHEC), encodes intimin, which is an outer membrane adhesin protein involved in the development of attaching and effacing (A/E) lesions by mediating the intimate adherence of these pathogens to enterocytes [59]. Although none of the examined APEC isolates carried the *stx1*, *stx2*, *STa*, *STb*, or *LT* genes, the presence of the *eae* gene might nonetheless pose a serious risk to human beings. Consequently, further comparative molecular investigations between *eae*-positive APEC isolates and human diarrheagenic *E. coli* (EPEC, EHEC) are warranted to assess the zoonotic potential of APEC.

In the current study, consistent with results from previous reports [48,60], genes encoding invasins, *yijP*, and *ibeB* were present in 98.98% and 97.96% of the APEC isolates, respectively. The high prevalence of the *yijP* gene in APEC isolates suggests that it may play a pivotal role in APEC pathogenesis by facilitating the bacterial traversal of the blood–brain barrier, as this gene is known to be involved in the invasion of human brain microvascular endothelial cells (BMECs) by *E. coli* [61].

Regarding the genes encoding protectins, the *ompA* and *iss* were identified in 97.96% and 79.59% of the APEC isolates, respectively. These data are consistent with previous research conducted in China [48].

The colicin V (ColV) plasmid operon genes *cva/cvi* were detected in 35.71% of the APEC isolates. Similar findings have been reported in APEC isolates from broiler breeders in Mississippi, USA [4]. The low prevalence of these genes among the tested isolates does not align with the high frequency of other ColV plasmid-related genes, namely, *iroN*, *iss*, *tsh*, and *iucD* [62]. This disparity may be due to alternative genetic carriers other than ColV plasmids, such as ColBM-type plasmids, or it may result from rearrangements or deletions within parts of the ColV operon [63].

The IrgA homolog adhesin (Iha), encoded by the *iha* gene, has been shown to contribute to the urovirulence of UPEC strains, participating in both adhesion and iron uptake as a catecholate siderophore receptor [64,65]. As part of this study, the *iha* gene was found in 8.16% of the APEC isolates, and despite being rare, its presence suggests a potential role in APEC pathogenesis and may be indicative of overlapping pathogenic strategies between human and avian ExPEC strains. Further studies are needed to investigate the contribution of Iha to APEC virulence and its potential utility as a diagnostic or epidemiological marker for high-risk strains posing threats to both poultry and human health.

Interestingly, among all isolates examined, a single isolate carrying both the capsule K1 biosynthesis (*neuC*) and invasion-related (*ibeA*) genes was identified within phylogenetic group B2. Although rare, this combination is noteworthy for its potential role in enhancing immune evasion and invasion capabilities, which may increase the systemic virulence of phylogenetic group B2 strains [12,66], as this group has been shown to demonstrate higher pathogenicity in experimental chicken infection models [51]. The presence of this gene combination in APEC, which is characteristic of human NMEC strains, particularly within phylogenetic group B2, suggests a possible zoonotic threat [28,67].

An experimental study demonstrated that deleting the yersiniabactin system genes, *fyuA* and *irp2*, from the APEC genome significantly reduced the transcription of virulence genes, decreased adherence to DF-1 cells (immortalized chicken embryo fibroblasts), and attenuated pathogenicity in chicks [68]. Furthermore, the vacuolating auto transporter toxin, encoded by the *vat* gene, has been shown to induce vacuolating cytotoxic activity in chicken embryonic fibroblasts and contributes to the development of cellulitis as well as the respiratory/systemic form of avian colibacillosis [69]. In addition to their role in avian hosts, it has been demonstrated that *E. coli* strains carrying a gene combination of *vat*, *fyuA*, *chuA*, and *yfcV* are capable of efficiently colonizing the urinary tract [70]. In the present study, among phylogenetic group F isolates, two distinct virulence profiles were observed, each represented by a single isolate, and both characterized by the simultaneous presence of *chuA*, *fyuA*, *irp2*, and *vat*. These virulence profiles correlated with isolates exhibiting the highest MVS. These results support previous observations indicating that isolates originating from phylogroup F may be highly virulent to chickens and may act as the primary cause of colibacillosis [71]. Moreover, *E. coli* of avian origin from phylogroup F has recently been the subject of research, suggesting its potential zoonotic risk [72]

In vitro antimicrobial susceptibility tests of the 98 APEC isolates showed alarming levels of resistance to ampicillin (97.95%), amoxicillin–clavulanic acid (96.93%), nalidixic acid (94.89%), tetracycline (90.81%), and ciprofloxacin (79.59%). In contrast, the resistance rates for trimethoprim–sulfamethoxazole (67.34%) and kanamycin (61.22%) were relatively moderate. These findings align with a prior report from Eastern Algeria, demonstrating high resistance to ampicillin, nalidixic acid, tetracycline, and ciprofloxacin [73]. The observed levels of antibiotic resistance are not surprising given the excessive and inappropriate use of these antimicrobial agents in the Algerian poultry sector. Additionally, the notable resistance of APEC to antibiotics considered critically important for human health, such as beta-lactams, fluoroquinolones, and tetracyclines, could potentially reduce their effectiveness in treating human infections [74].

Resistance to cefotaxime (17.34%) is lower than previously reported in Algeria and Qatar [73,75]. Even though third-generation cephalosporins have not been used in Algerian poultry production systems, the occurrence of extended-spectrum cephalosporin-resistant *E. coli* presenting the extended-spectrum beta-lactamase (ESBL)-producing phenotype has been documented previously [20]. In addition, ESBL genes, including *bla*TEM-141 and *bla*CTX-M-55, carried on conjugative plasmids (IncF-type), can be horizontally transferred between *E. coli* and related species such as *E. albertii* [76]. From a One Health perspective, this horizontal gene transfer represents a potential risk to human and animal health, as these plasmids can disseminate resistance across bacterial populations in animals, humans, and the environment [77]. Continuous monitoring of plasmid-mediated resistance in livestock is therefore essential to anticipate zoonotic transmission and to guide antimicrobial stewardship strategies.

In this study, a low resistance level to gentamicin (20.41%) was observed. This finding aligns with results described in prior studies in Algeria and Turkey [21,78]. The noted resistance to gentamicin, despite being at low levels, may be attributed to its unofficial use, knowing that this antimicrobial has been banned for veterinary use in Algeria.

The resistance rate to chloramphenicol in our study was relatively low (21.43%). This is in agreement with findings from other researchers who have reported comparable levels of resistance [20,73]. Despite chloramphenicol being prohibited in Algeria since 2006 [79], some *E*. *coli* strains remain resistant, probably due to co-resistance, which occurs as a result of the frequent use of other antibiotic molecules. Reports have indicated that chloramphenicol co-resistance in *E. coli*, isolated from diseased cattle and swine, can arise from the use of other antibiotics such as ampicillin, dihydrostreptomycin, kanamycin, trimethoprim, sulfamethoxazole, and tetracycline [80,81].

In addition to resistance to each antibiotic, our findings indicated that 92.86% of the APEC isolates were multidrug-resistant (MDR), highlighting a significant concern. This is consistent with previous studies from Algeria (92%) [82], Tunisia (96%) [83], and China (100%) [48]. Indeed, the arsenal of effective antibiotics available for treating APEC infections has become increasingly limited, leading to a therapeutic impasse.

## 5. Conclusions

To the best of our knowledge, this work reports, for the first time, the potential emergence of O157 as one of the predominant serotypes of APEC in Algeria. This finding may indicate the possible existence of other serotypes associated with avian colibacillosis that have not yet been identified.

Additionally, the APEC isolates examined were predominantly assigned to phylogenetic groups B1 and C. These isolates presented a notable prevalence of various virulence genes commonly associated with the ExPEC pathotype, as well as high antibiotic resistance. Furthermore, our findings demonstrated that some APEC isolates from phylogenetic groups B2 and F share key virulence determinants with human ExPEC. These APEC may exhibit high virulence in avian hosts and could either act as human ExPEC or serve as a reservoir for their virulence genes. However, further investigations with a larger sample size are required to evaluate the extent of this genetic overlap, as the number of APEC isolates identified in phylogenetic groups B2 and F in this research was relatively low.

These findings underscore the importance of continuous surveillance of these pathogens in poultry flocks and the need for judicious antibiotic use to ensure effective management of APEC infections and mitigate the risk of zoonotic transmission.

## Figures and Tables

**Table 1:** Distribution of virulence genes based on APEC serotypes.

Virulence Genes	O157	O78	O8	O9	Unidentified Serotypes
	*n* (%)	*n* (%)	*n* (%)	*n* (%)	*n* (%)
*crlA*	20 (100)	11 (100)	2 (100)	1 (100)	64 (100)
*mat*	20 (100)	11 (100)	2 (100)	0 (0)	64 (100)
*yijP*	20 (100)	11 (100)	2 (100)	1 (100)	63 (98.44)
*ibeB*	20 (100)	11 (100)	2 (100)	1 (100)	62 (96.88)
*fimC*	18 (90)	11 (100)	2 (100)	1 (100)	64 (100)
*ompA*	20 (100)	11 (100)	2 (100)	1 (100)	62 (96.88)
*iucD*	18 (90)	11 (100)	2 (100)	1 (100)	56 (87.50)
*iroN*	18 (90)	9 (81.82)	2 (100)	0 (0)	51 (79.69)
*iss*	15 (75)	9 (81.82)	2 (100)	1 (100)	52 (81.25)
*eae*	16 (80)	8 (72.73)	2 (100)	1 (100)	51 (79.69)
*tsh*	8 (40)	0 (0)	1 (50)	0 (0)	46 (71.88)
*fyuA*	8 (40)	4 (36.36)	1 (50)	0 (0)	39 (60.94)
*irp2*	8 (40)	4 (36.36)	1 (50)	0 (0)	33 (51.56)
*cva/cvi*	10 (50)	4 (36.36)	1 (50)	1 (100)	19 (29.69)
*aatA*	7 (35)	0 (0)	0 (0)	0 (0)	20 (31.25)
*chuA*	2 (10)	0 (0)	1 (50)	0 (0)	11 (17.19)
*iha*	5 (25)	0 (0)	0 (0)	0 (0)	3 (4.69)
*neuC*	2 (10)	0 (0)	0 (0)	0 (0)	0 (0)
*ibeA*	1 (5)	0 (0)	0 (0)	0 (0)	1 (1.56)
*vat*	0 (0)	0 (0)	0 (0)	0 (0)	2 (3.13)

**Table 2:** Distribution of virulence genes within the identified APEC phylogenetic groups.

Virulence Genes	No. (%) of APEC Isolates by Phylogenetic Group					
	A (*n* = 12)	B1 (*n* = 43)	B2 (*n* = 2)	C (*n* = 29)	E (*n* = 7)	F (*n* = 5)
*crlA*	12 (100)	43 (100)	2 (100)	29 (100)	7 (100)	5 (100)
*mat*	12 (100)	43 (100)	2 (100)	29 (100)	7 (100)	5 (100)
*yijP*	12 (100)	42(97.67)	2 (100)	29 (100)	7 (100)	5 (100)
*ibeB*	12 (100)	41(95.35)	2 (100)	29 (100)	7 (100)	5 (100)
*fimC*	11 (91.67)	43 (100)	2 (100)	28 (96.55)	7 (100)	5 (100)
*ompA*	12 (100)	43 (100)	2 (100)	29 (100)	6 (85.71)	4 (80)
*iucD*	11 (91.67)	38 (88.37)	2 (100)	25 (86.21)	7 (100)	5 (100)
*iroN*	10 (83.33)	37 (86.05)	2 (100)	23 (79.31)	5 (71.43)	3 (60)
*iss*	7 (58.33)	36 (83.72)	1 (50)	27 (93.10)	4 (57.14)	4 (80)
*eae*	9 (75)	37 (86.05)	1 (50)	21 (72.41)	6 (85.71)	4 (80)
*tsh*	8 (66.67)	19 (44.19)	1 (50)	19 (65.52)	4 (57.14)	4 (80)
*fyuA*	5 (41.67)	25 (58.14)	1 (50)	16 (55.17)	0 (0)	5 (100) ^b^
*irp2*	3 (25)	23 (53.49)	0 (0)	16 (55.17)	0 (0)	4 (80) ^b^
*cva/cvi*	1 (8.33)	28 (65.12)	1 (50)	2(6.90)	2 (28.57)	1 (20)
*aatA*	6 (50)	12 (27.91)	2 (100)	1(3.45)	3 (42.86)	3 (60)
*chuA*	0 (0)	0 (0)	2 (100)	0 (0)	7 (100)	5 (100) ^b^
*iha*	1 (8.33)	4 (9.30)	1 (50)	0(0)	0 (0)	2 (40)
*neuC*	1 (8.33)	0 (0)	1 (50) ^a^	0 (0)	0 (0)	0 (0)
*ibeA*	0 (0)	1 (2.33)	1 (50) ^a^	0 (0)	0 (0)	0 (0)
*vat*	0 (0)	0 (0)	0 (0)	0 (0)	0 (0)	2 (40) ^b^
MVS ^c^	11.08	11.97	13.50	11.13	11.28	14.20

^a^ The simultaneous presence of the *neuC* and *ibeA* genes. ^b^ The *vat*, *fyuA*, *irp2*, and *chuA* genes were present simultaneously in the same isolates. ^c^ MVS (the sum of all virulence genes found in isolates in a given group/number of isolates in that group).

## Data Availability

The data presented in this study are available within the manuscript.

## Supplementary Materials

The following supporting information can be downloaded at https://www.mdpi.com/article/10.3390/ani15223324/s1, Table S1: PCR primers used to detect virulence and O-antigen genes in APEC; Table S2: Serotyping results of APEC isolates; Table S3: Distribution of phylogenetic groups among APEC isolates; Table S4: Frequencies of virulence genes in the 98 APEC isolates; Table S5: Prevalence of sensitive, intermediate, and resistant APEC isolates; Table S6: Prevalence of multidrug resistance (MDR) in APEC isolates; Table S7: Details of the APEC isolates.
